# Lateral Flow Assay: A Summary of Recent Progress for Improving Assay Performance

**DOI:** 10.3390/bios13090837

**Published:** 2023-08-23

**Authors:** Kobra Omidfar, Fatemeh Riahi, Soheila Kashanian

**Affiliations:** 1 Biosensor Research Center, Endocrinology and Metabolism Molecular—Cellular Sciences Institute, Tehran University of Medical Sciences, Tehran 1458889694, Iran; 2 Endocrinology and Metabolism Research Center, Endocrinology and Metabolism Research Institute, Tehran University of Medical Sciences, Tehran 1458889694, Iran; 3 Faculty of Chemistry, Razi University, Kermanshah 6714414971, Iran; 4 Nanobiotechnology Department, Faculty of Innovative Science and Technology, Razi University, Kermanshah 6714414971, Iran

**Keywords:** lateral flow assay, principle, performance improvement

## Abstract

Lateral flow tests are one of the most important types of paper-based point-of-care (POCT) diagnostic tools. It shows great potential as an implement for improving the rapid screening and management of infections in global pandemics or other potential health disorders by using minimally expert staff in locations where no sophisticated laboratory services are accessible. They can detect different types of biomarkers in various biological samples and provide the results in a little time at a low price. An important challenge regarding conventional LFAs is increasing their sensitivity and specificity. There are two main approaches to increase sensitivity and specificity, including assay improvement and target enrichment. Assay improvement comprises the assay optimization and signal amplification techniques. In this study, a summarize of various sensitivity and specificity enhancement strategies with an objective evaluation are presented, such as detection element immobilization, capillary flow rate adjusting, label evolution, sample extraction and enrichment, etc. and also the key findings in improving the LFA performance and solving their limitations are discussed along with numerous examples.

## 1. Introduction

Lateral flow assays (LFAs), as a mature strategy in the area of paper-based POC devices, are the cheapest, fastest, and easiest to use paper-based assays. They are utilized as an efficient test for the on-site analysis and quantification of biomarkers, such as proteins, small molecules, nucleic acid, etc., from a variety of different biological samples, including blood, urine, saliva, and many other types [1,2,3,4,5,6]. The home-made pregnancy test is the first paper-based LFA assay which can distinguish the level of human chorionic gonadotropin hormone in urine samples [7]. 

**Figure 1:**
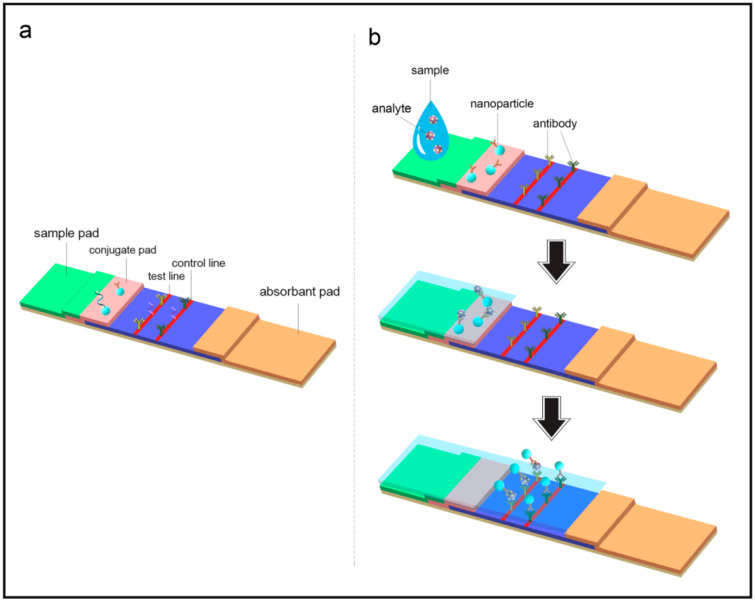
(**a**) A typical diagram and several components of an LFA. (**b**) Start the assay by adding a liquid sample, in which target analytes specifically bind to the nanolabeld detection probes. With the flow of the sample, the analyte-nanolabels probe complex binds to the capture probe at the test line (positive result) and control line (proof of validity), respectively.

Conventional LFAs comprise several elements, including a sample pad, a conjugate pad, a reaction membrane, and an absorbent pad that are assembled together and are supported by a plastic backing card (Figure 1a). In LFA, a liquid sample comprising the target analyte moves through test and control lines of paper strips under capillary force and makes contact with the capture probe immobilized on the membrane (Figure 1b). LFAs require a small sample volume to generate qualitative, semi-quantitative, or quantitative results within 5–15 min, which is almost always related to an optical signal produced at the test line [8,9,10,11]. Qualitative analysis refers to a screening or preliminary assay that defines the presence or absence of a target analyte in a sample.

For example, in pregnancy tests and the detection of pathogenic bacteria and viruses, a simple binary readout is enough to show whether the test is either positive or not. For instance, Jiang et al. [12]. reported the fabrication of a microfluidic paper-based device in combination with nitrocellulose membrane as the protein immobilization substrate and cellulose paper as the water absorption substrate for the detection of two bladder cancer biomarkers, including nuclear matrix protein 22 (NMP22) and bladder cancer antigen (BTA) from urine samples. To reduce the cost and simplify the fabrication, wax printing was employed for the construction of a chip and colloidal gold immune labeling was also applied for direct result observation by the naked eye. In another study by Ahmadi et al. [13], a qualitative lateral flow immunoassay (LFIA) was established for the simultaneous recognition of IgM/IgG antibodies specific to severe acute respiratory syndrome coronavirus 2 (SARS-CoV-2). This LFIA, based on the labeling of recombinant SARS-CoV-2 antigens with colloidal gold nanoparticles, was capable of detecting the IgM/IgG antibodies specific to the virus in 20 µL of serum or plasma samples with a sensitivity of 90% and specificity of 96.6%. Semi-quantification relies on identifying an approximate amount of analyte via visual evaluation, without the use of an external tool. The results are often expressed as the analyte concentration level in a sample into high (+++), medium (++), low (+), and very low or absence (−) based on the color intensity of the test line. Petrou et al. [14] developed an LFA test strip integrated with automated sample processing for semi-quantitative analyses of microRNA-150-5p from human plasma exosome, a predictive biomarker for preterm birth (PTB). Peptide nucleic acid (PNA) probes were used to determine microRNA quantitatively via oligonucleotide-templated reaction (OTR). After the addition of plasma to the denaturing hydrogel composite sample pad, the exosomal microRNA-150-5p released and bound to peptide nucleic acid (PNA-1), which was immobilized on the nitrocellulose membrane under the sample pad, then moved along the membrane and captured by PNA-2 at the test line. This assay could recognize the levels of microRNA from only three microliter of sample in 45 min and discriminate between patients at low and high risk of PTB with a *p*-value less than 0.0001.

Quantitative examination is described as the ability to determine the exact amount of an analyte in a sample environment and express it as a numerical value in appropriate units, which can be performed via the use of reader systems with simple operation processes. The validations of analytical methods and qualitative analysis are required to achieve a reliable calibration curve. Most commercially available LFAs offer qualitative or semi-quantitative analyses and require dedicated tools for the quantitative analysis of biomarkers. Wu et al. [15] developed an LFA strip for the rapid and quantitative measurement of brain-derived neurotrophic factor (BDNF) concentration in artificial tear fluids. The results of the test were assessed by utilizing a portable and inexpensive device, including a smartphone camera attached to a black readout box. This assay showed that reliable quantitative results in the range of 25 to 300 pg mL^−1^ with a detection limit of 14.12 pg mL^−1^. Whereas commercial LFAs are very valuable methods, they are still restricted to the qualitative or semi-quantitative determination of one highly concentrated analyte, lacking the sensitivity required for the identification of the lowest amount of analyte, in particular concerning the complex biological samples. LFAs also have low resolution, are unable to control the flow rate, and require a large volume of reagents. To improve the sensitivity of the LFA and obtain high sensitive accurate tests, many efforts have been devoted to addressing these limitations. Two significant procedures for increasing the sensitivity are assay improvement and target augmentation of a limited sample. These approaches can increase the sensitivity of analysis by several times and also facilitates multiplexing determinations of various biomarkers in complex media. Assay specificity is also significant and strongly relies on the affinity, stability, and properties of the immobilized capture molecule. It is typically enhanced through the use of capture and detection reagents such as antibodies and nucleic acid probes (often DNA because of greater stability than RNA) with better affinity and/or avidity and higher stabilities for the target molecules as well as assay optimization to reduce non-specific reactions [2,16,17]. The usage of novel capture and detection molecules such as nanobodies, short peptides, and aptamers can increase the specificity of the assay [2,18,19]. Small molecules like nanobodies have longer antigen-recognizing domains, enabling their reach to target analytes unreachable to full antibodies. They can detect, bind, and target small epitopes that are inaccessible for full antibodies [18,20,21]. Good accessibility and functionality of the surface-bound elements, which closely relate to the chemistry of attachment, is another feasible strategy to improve the specificity of the assay. The configuration of the recognition element can also affect the performance of the assay [22,23].

In this study, we summarized the most very recent significant concepts (from 2021 to 2023) related to the enhancement of assay performance, especially LFA, and attempted to report the most effective and applicable findings. Each improvement strategy is discussed based on the configurations of main component parts such as the amplification mechanism, recognition elements, and design.

## 2. Methodology

This study focused on recent reports published in peer-reviewed journals indexed in Web of Science, ScienceDirect, Scopus, PubMed, and Google Scholar. The following keywords were used for searching: sensitivity enhancement or improvement in lateral flow assay, specificity improvement in lateral flow assay, signal amplification methods, sensitivity and specificity improvement in point of care testing, nanomaterial application in lateral flow assay, increasing immobilization efficiency in the assay, and lateral flow assay and disease marker. Ninety-one papers were identified from 180 articles. They were reviewed in order to critically address issues on sensitivity enhancement or improvement in the assay, specificity improvement in the assay, lateral flow assay development, signal amplification methods, and highlighting the trends and challenges for analyzing the low concentration of analytes in real samples. Table 1 summarizes various sensitivity and specificity improvement approaches reported for LFA.

## 3. Biorecognition Strategies

Antibodies are very common biorecognition components in LFA, mostly due to their excellent selectivity, good binding activity, production, and commercial availability. 

However, in recent years, nanobodies and nucleic acid probes have also been able to serve as possible alternatives to antibodies in LFA due to their remarkable advantages [43,44]. When nanobody, antibody, and antibody derivatives, including fragment antigen binding (Fab) or single chain variable fragments (scFv), are utilized as biorecognition components, this assay is known as LFIA. Van der Waals, non-polar hydrophobic interactions, London dispersion attractive forces, and steric repulsion forces are non-covalent forces that commonly contribute to establishing molecular interactions between the antibody and antigen. Oligonucleotide probes are other biorecognition components that are usually employed for the recognition of their complementary target sequences. Low sensitivity is one important limitation of present nucleic acid-based LFAs which restricts its direct recognition after nucleic acid extraction and purification. Therefore, target sequences are usually pre-amplified, then termed DNA amplicons, before use in an assay. Molecular beacons are a class of nucleic acid probes with single-stranded DNA hairpin structures which can be employed as biorecognition elements in LFA. Peptide nucleic acids (*PNA*) are another class of nucleic acid analog that have received particular interest as alternatives to DNA probes, especially for short nucleic acid sequences. They are synthetic single-strand analogs of DNA/RNA in which the sugar-phosphate backbone has been replaced by a peptide backbone [43]. LFA combined with PNA has successfully improved SARS-CoV-2 identification [45]. Aptamers, also known as chemical antibodies, are short sequences of artificial DNA, RNA, XNA, or peptide that have the ability to bind to a wide range of target analytes. They have 2-dimensional or 3-dimensional structures, which enables them to bind selectively to target molecules with high affinity through a variety of binding interactions, including Van der Waals, hydrogen bonds, electrostatic, aromatic ring stacking, salt bridges, and shape complementarity [2,43].

## 4. Assay Improvement

Recent studies to enhance analytical performance through assay improvement comprise assay optimization by either increasing the reaction time or increasing the reactant concentration and signal amplification by new chemical/physical modifications, new label design, and reader use. Another important issue is the way in which the recognition element and the surface of the main components are configured, as both will significantly affect the performance of the assay.

### 4.1. Assay Optimization

Assay optimization is an essential part of LFA establishment and is necessary for improving LFA sensitivity. Assay optimization (i.e., the transport and reaction conditions) concerns experiments that determine how a range of parameters and conditions affect assay performance.

#### 4.1.1. Controlling Capillary Flow Rate

Adjusting the capillary flow rate of samples and reagents in LFAs is of great importance because the flow rate can influence the reagent's dissolution and mixing as well as the effectiveness of reactions. Reaction and transport are indissolubly related in that alterations to transport properties such as volumes or flow rates automatically alter reaction kinetics. One of the key reasons for the poorer sensitivity of LFAs in comparison to other standard laboratory tests, such as the enzyme-like immunosorbent assay (ELISA), is that they do not permit as much time for the specific binding of biorecognition receptors to the antigens or target biomarkers. This is due to the inherent capillary effect of paper fibers (also known as wicking), which creates a unidirectional flow of the liquid solution at a constant value across the membrane.

Numerous fluidic controls have been established to regulate transport dynamics, adjust reaction kinetics, and enhance sensitivity in paper-based POC devices. Ideally, the sample would be kept on the test region to augment attaching of target analytes and labeling detectors to capture molecules at the test region and consequently enhance assay sensitivity. Various methods have been established in recent years to prepare an environment that permits the improved attaching of target analytes and labeling detectors to capture molecules at the test region. Reducing the flow rate in LFAs can be accomplished by altering the geometry of the strip components or by chemically modifying the nitrocellulose membrane; therefore, due to the lower flow rate of sample and reagents within the paper strip, target analytes have further time to interrelate with the capture and nanolabel probes. Modified paper geometry can be achieved by employing a sample pad, conjugate pad, or nitrocellulose membrane shape and pore size alterations [24,25].

For example, introducing slight angles to the input and output in the lateral flow device creates a barrier in the path of the flowing fluid to control wicking times (the flow rates) and increase the time of interaction of biomolecules for controlling and optimizing the binding of the implemented test. Iles et al. introduced a geometric flow control LFIA based on decreasing test line width by placing a photo-sensitive polymer onto the nitrocellulose film. They utilized a laser to polymerize and create an impermeable barrier across the depth of the film. They showed that both the location and the number of constrictions can affect the sensitivity of the assay due to a slower capillary flow rate within the test strip and a higher quantity of specimens per unit width of the test region. By adding two constrictions, they achieved a 62% improvement in test line color intensity for the recognition of procalcitonin (PCT). They reduced the limit of detection (LOD) from 10 ng/mL to 1 ng/mL. The authors pointed out that this strategy can remarkably reduce the antibody consumption per device, resulting in lower construction costs of tests [25]. Li et al. established a ball pen writing-without-ink technique to adjust fluid flow and augment the detection sensitivity of LFAs by making changes in the porosity and pore size of the paper. Authors obtained a two-fold increase in detection sensitivity using presented approch. [24].

Chemical flow control can be carried out by inserting flow-interrupting materials in the LFA strip to decrease the flow rate. An example includes the incorporation of salt, sugar, wax, polymer, etc., on the paper to modify flow rates and decrease flow time [46,47,48]. Recent works in the area have focused on utilizing polymeric chemical compounds as barriers. For example, the insertion of aerogels or agar and polymeric nanofibers into a nitrocellulose membrane after the conjugate pad or between the conjugate pad and the nitrocellulose membrane can decrease the sample flow rate in the LFA and enhance the detection signal intensity [26,49]. In a study by Park and Shin, a LFA strip was developed by applying pressure on the top of nitrocellulose film between the test line and the control line to decrease the flow rate and increase the signal intensity. They reported a two-fold sensitivity improvement for the recognition of C-reactive protein compared to a conventional, non-pressed LFA [26].

Another group reported a delayed LFA, which comprises two separate layers. The upper membrane was utilized wholly for the flow path of the target molecule. The lower layer was fabricated using a hydrophobic trimethylsilyl cellulose (TMSC) wall on the bottom of the conjugate pad to time-delay the movement of the SARS-CoV-2 spike receptor-binding domain (SARS-CoV-2 SP RBD) conjugated to a gold nanoparticle. This hydrophobic layer reduced the movement of the labeled SP RBD to the specific antibody on the test spot, thus allowing the labeled antigen to react with the specific antibody effectively. Their method resulted in a 2.6-fold sensitivity improvement for SARS-CoV-2 SP RBD detection and 9.1-fold LOD enrichment in comparison to the conventional LFIA [27]. To control the capillary flow rate in the lateral flow device, Lee et al. presented a delaminating timer by imprinting barricades on the path of flow using water-insoluble ink and employing the gradual creation of a void among a wetted paper and a sheath polymer tape. The authors achieved an eight-fold enhancement in the LOD for human chorionic gonadotropin measurement compared to the conventional LFA [28]. Other published works in this field employed cellulose fibers as the sample pad, creating a wall before the test line region onto the nitrocellulose film, and the use of chitosan and nanofiber on nitrocellulose membrane [29,50,51]. All these approaches can slow the flow rate and modify the sensitivity of recognition from one to multiple folds in LFA. However, some of these procedures require either unusual or complex construction methods limiting their use in practice or large-scale production.

#### 4.1.2. Immobilization Efficiency of Biorecognition Elements

One of the main factors of biosensing device development is the selection of a suitable immobilization method. Loading density, orientation of immobilized detection elements, reaction surfaces, and ligand-binding efficiency following their immobilization onto the functionalized surfaces are the main elements that strongly affect the analytical performance of the assay. This is important to immobilize detection elements onto the reaction surfaces without altering their structure, function, binding activity, and specificity. Therefore, these approaches should be mild and compatible with the various components of the system and also provide high performance in the developed assay. Non-covalent binding, including passive immobilization by adsorption forces, electrostatic interactions, and affinity reactions, as well as covalent binding, are various methods which have been utilized for immobilizing detection elements onto support surfaces [2,17,22,52,53,54]. The passive immobilization method provides the most direct and simple approach, but it is relatively random and uncontrolled. The covalent immobilization method is typically formed between the available functional groups that are located on the surface of the detection element and the properly-modified reaction surface. They produce effective and irreversible attachment with good stability and high binding strength and also control the orientation and density of detection elements. However, covalent immobilization has several limitations, such as requiring linker molecules, exhibiting a slow process and crowding effects. The best immobilization technique should be selected based on the detection element chosen, the support surface, the physical–chemical atmosphere and also the analyte characteristics [22,52,55]. Increasing the efficiency of capture molecule immobilization onto the support surface is another practical or powerful approach to enhance sensitivity and improve analytical detection, as this will lead to a higher analyte binding efficacy. However, this method is restricted due to the immobilization capacity of the biomolecule on the support surface. Many efforts have been applied in order to enhance the immobilization density of affinity biomolecules (biorecognition elements) on the support nature in assays. Nanomaterials are among particular compounds ranging from zero to three-dimensionality and have a high surface-to-volume ratio introduced to increase the immobilization efficacy of biomolecules and the analytical sensitivity of an assay [56,57,58,59,60,61].

In LFA devices, nanomaterials are utilized to reach three goals: functionalization of the nitrocellulose membrane surface and efficient immobilization of the bioactive compounds, increasing the active surface area for augmenting the number of immobilized elements, and employing as carriers to load signal tags or directly as signal reporters for enhancing the optical signal. For example, Omidfar et al. developed a competitive LFIA to detect human serum albumin (HSA) in urine samples with the use of antibody-functionalized gold nanoparticles as capture probes. HSA was also attached to mobile crystalline material (MCM)-41 nanoparticles and coated onto nitrocellulose film in the test region. In this study, the MCM-41-type silica nanoparticle was applied for efficient attachment of the capture molecule onto the nitrocellulose membrane at the test region. The authors achieved higher signal intensity at the test line in the presence of MCM-41 than conventional LFIA [62,63].

Another group reported a fluorescence-based LFIA for the quantification of traumatic brain injury (TBI) using ubiquitin carboxyl-terminal hydrolase-L1 (UCH-L1) as a biomarker and graphene oxide (GO) particles over the test region of nitrocellulose membrane as immobilization support. Experimental results showed that the addition of 80 ng of GO at test regions improved fluorescence signals 2–3-fold. GO can enhance the capture of antibody immobilization efficiency at the test region, resulting in an augmented density of recognition complexes on the test region surface and hence the final signal [30].

Recently, electrospun nanofibers have been introduced as a substrate for fabricating or modifying nanoscale POC-based biosensors due to their large surface area-to-volume ratio, tunable chemical surfaces, and high porosity [57,59,64]. As an example, modifying the membrane with nitrocellulose nanofibers to adjust the porosity for boosting the protein load and slowing flow rate can also increase the detection sensitivity by 50-fold [29].

The orientation of immobilized recognition elements is also a key parameter affecting the analytical performance of the assay. Orientation of detection elements onto sensing platforms increases the overall antigen-binding capacity in assays, and this contributes to improving the sensitivity and specificity of them. Assay performance improvement by orienting biological elements with protein A, thiol group functionalization, and streptavidin–biotin interaction has been revealed in numerous works once compared to their randomly immobilized counterparts [22,53]. In a study by Yang et al., a cellulose membrane-based LFIA was established using CBP31-BC fusion composed of cellulose-binding domains (CBDs) and antibody-binding B and C domains of protein A. Owing to oriented antibody attachment on cellulose by this linker, the presented LFIA showed almost 10-fold higher sensitivity to prostate-specific antigens than nitrocellulose membrane-based conventional LFIAs [31]. In another study by Lee et al., the same linker (CBP31-BC) was utilized to immobilize antibodies on a cellulose membrane film in an oriented mode. The developed LFIA was utilized for the detection of SARS-CoV-2. The clinical performance of the assay for detecting SARS-CoV-2 was evaluated using 19 clinical samples. This LFIA was capable of detecting SARS-CoV-2 with 100% accuracy [22].

### 4.2. Signal Amplification

A simple method to improve the measurable signal is enhancing the positive test region colorimetric contrast. Stronger contrast can be accomplished by changing the structure and size of the gold nanoparticles via chemical/physical modifications or by substituting gold nanoparticles with particle clusters or particles prepared by other metals, metal oxides, or organic materials.

***New Chemical/Physical Modifications*:** One approach to enhance the sensitivity of LFA is the surface modification of gold nanoparticles. In a study by Xu et al., polydopamine-coated gold nanoparticles were prepared by oxidative self-polymerization of dopamine on the surface of nanoparticles. The result showed that the detection limit of modified label gold nanoparticles-based LFA was ten-fold lower than that of traditional LFA [65]. Similarly, label materials coated on silica surfaces can reduce the background signal by imparting high particle stability, amplifying the signal intensity, and also increasing the surface area, which enables the loading of more biorecognition elements. Kim et al. coated Au–Ag alloy nanoparticles on silica surfaces to increase the signal intensity of LFA for detecting blood prostate-specific antigen (PSA) levels in clinical samples. The LOD for PSA by this modification was lowered to 0.30 ng mL^−1^ [32].

There are also several strategies to augment the plasmonic signal of gold nanoparticles through color change at the test region using chemical reactions. The most important procedure is gold nanoparticle enlargement through silver or copper staining, loading with enzymes or catalytic metals. In the copper and silver enhancement technique, silver and copper ions are reduced to silver and copper around the gold nanoparticles in the test line by utilizing reducing agents through an LFA after a normal assay. The resulting copper and silver layer on the surface of gold nanoparticles enhances the color intensity of the test region. As an example, using gold nanoparticle-induced copper deposition achieved a three-fold lower LOD in the detection of rabbit IgG compared to a conventional test strip without any signal amplification [66].

Nanozymes are nanomaterials with enzyme-mimicking ability. They have the unique characteristics of nanomaterials and natural enzyme-like catalytic activities. The use of nanozymes as labels in LFA has recently been developed [33,44]. For example, an LFA was developed by Scarsi et al. for the assessment of total antioxidant capacity (TAC) in saliva samples by employing the nanozyme characteristics of platinum nanoparticles (PtNPs). This multi-line catalytic platinum-based LFA showed a semi-quantitative analysis of TAC within 10 min through the naked-eye or a smartphone readout [33].

***New Label Design*:** The label is one of the substantial elements of LFA for the creation of a signal. Detection labels are stable materials under standard storage and various conditions of experiments. They have low or no non-specific background binding and can be easily attached to biomolecules or chemical elements. Gold nanoparticles and colored latex beads are widely utilized as labels for the generation of visible signals in most commercial LFA. However, they often have limited detection sensitivity when low concentrations of analyte need to be detected, so it is necessary to establish new label design procedures with high sensitivity and low cost. An alternative procedure for augmenting LFAs sensitivity is to utilize detection labels with higher absorbances that produce a stronger contrast with the background signal [34,35,36,37]. Song et al. developed a surface-enhanced Raman scattering (SERS)-based LFA for cardiac troponin I measurement by conducting three optimization procedures. The authors achieved a stronger SERS intensity by optimizing the size of gold nanoparticles in SERS tags, lowering reaction time, and enhancing LFA buffer components. The sensitivity of SERS-based LFIA prepared by this technique was 78-fold larger than that of the optical sensing method [34]. Li et al. established and developed a SERS-based LFA system, integrated with a catalytic hairpin assembly (CHA) amplification approach, for the detection of miR-106b and miR-196b related to laryngeal squamous cell carcinoma (LSCC). By introducing target probes, the modified biotin on the surface of palladium-gold core–shell nanorods was exposed by two hairpin DNAs self-assembling into double-stranded DNA and captured using streptavidin which was immobilized on the test region. Their method could amplify the SERS signal, and they achieved a limit of detection around the aM level [35].

Compared with colorimetric labels, LFAs based on fluorescent molecules or nanoparticles as reporter elements have the advantages of high sensitivity, good stability, and quantitative ability. Over the past decade, different fluorescent nanoparticles have been studied and developed to enhance the sensitivity of LFAs. Quantum dots (QDs) are among the most used labels owing to their narrow emission peaks, wide absorption cross-section, strong fluorescence emission intensity, and high quantum yields. Recently, Wang et al. developed a fluorescence LFA strip for the detection of SARS-CoV-2-specific IgM/IgG in clinical samples by utilizing S protein-conjugated SiO_2_@dualQD nanotags. This fluorescent core/shell label was fabricated using a monodisperse SiO_2_ core with a 200 nm diameter which was coated by a dual layer of carboxylated QD shell. The reported SiO_2_@DQD-based LFA was able to detect a low concentration of IgM/IgG (1:10^7^ dilution) from 1 μL serum within 15 min [67]. In another example, Huang et al. developed a pitaya-type silica porous material loaded with high-density QDs as a label for the enrichment of signal *strength* in LFA. The presented LFA could measure C-reactive protein levels in clinical samples with a linear range from 0.125 to 300 ng/mL [68].

**Figure 2:**
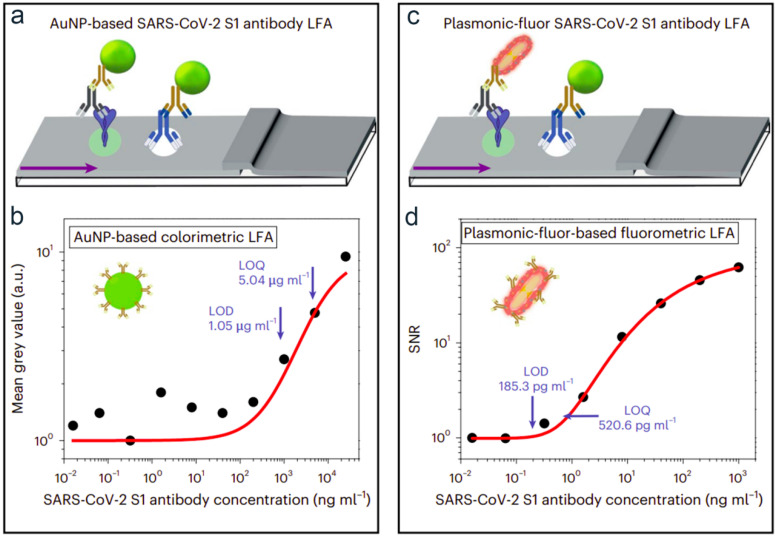
(**a**,**c**) Schematic view of the SARS-CoV-2 S1 antibody LFA tests, including the recombinant S1domain antigen of the SARS-CoV-2 spike protein as a capture probe at the test line and sheep immunoglobulin G at the control line. (**a**) A diagram of AuNP-based SARS-CoV-2 S1 antibody LFA. (**c**) A diagram of plasmonic-fluor-based SARS-CoV-2 S1 antibody LFA. Dose-dependent mean gray values (**b**) and dose-dependent SNR ratio (**d**) of the SARS-CoV-2 S1 antibody obtained from AuNP and plasmonic-fluor-based LFA, respectively. Reprinted (adapted) with permission from Ref. [36]. Copyright 2023 Springer Nature.

In recent years, other alternative labels have also shown promise, particularly the use of the plasmonic construct by Gupta et al. The plasmonic construct consists of a bovine serum albumin scaffold covalently attached to fluorophores and biotin and later covered around the plasmonic nanomaterials. The fluorescent emission intensity of these nanocomposites, by changing particle size, shape, and composition, can be tuned from the visible to near-infrared spectral regions. The authors examined the bioanalytical performance of gold nanoparticles LFA (Figure 2a) and plasmonic LFA for the detection of the SARS-CoV-2 S1 antibody (Figure 2c). The limit of detection of gold nanoparticles-based LFA was around 1.05 μg mL^−1^ (Figure 2b). In contrast, with the plasmonic approach, the limit of detection was 185 pg mL^−1^, which represents a nearly 5675-fold improvement (Figure 2d) [36].

**Figure 3:**
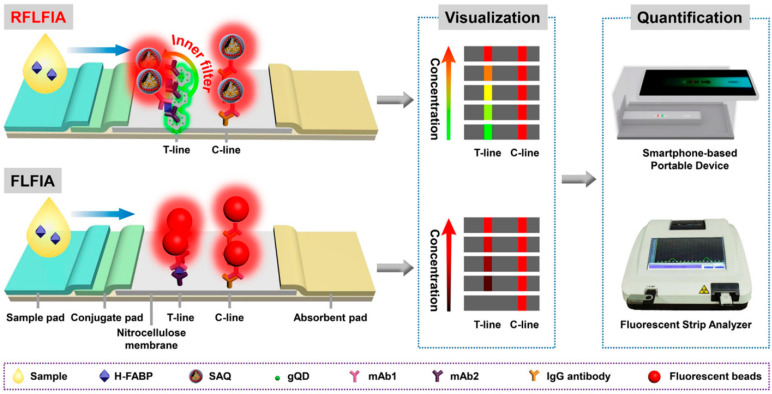
Schematic view of ratiometric and conventional fluorescence LFA (RFLFA and FLFA), respectively, for visual and quantitative analysis of H-FABP. Reprinted (adapted) with permission from Ref. [37]. Copyright 2021 Wiley.

Wang et al. established a sensitive ratiometric fluorescent LFA for the quantitative and visual detection of heart-type fatty acid binding protein (H-FABP). A silica nanosphere loaded with gold nanoparticles and red-light emitting CdSe/CdS/ZnS QDs (rQDs) was used as a tracing tag to label the primary antibody; meanwhile, green-light emitting CdZnSe/CdS/ZnS QDs (gQDs) conjugated to the secondary antibody were immobilized in the test region. The results of the assay were analyzed using an inexpensive smart reader containing a three-dimensional printed compact attachment and a smartphone. A limit of detection as low as 0.21 ng mL^−1^ was achieved, which is more accurate and sensitive than conventional fluorescent LFA with only a single-label response (Figure 3) [37].

***Reader Use*:** LFA sensitivity can also be augmented via employing smart readers because the signal intensity is particularly relative to the number of captured particles in the test zone, which is also related to the quantity of biomarkers in the sample. With readers, external stimuli, including light, electric potential, or magnetic field, can excite captured nanoparticle labels in the test zone to create an augmented signal. This signal is then identified using external sensitive optical/electrical/magnetic devices that can differentiate slight signal alterations through non-specific binding and amplify the detection sensitivity by several times over the conventional naked-eye readout [69,70]. Recent progress has resulted in the establishment of software and apps as well as several types of readers based on the excitation technique such as fluorescence, SERS, thermal, magnetic amplification, and electrochemistry. These systems should be designed to be portable, affordable, low-cost, and sensitive to on-site analysis. Because of the considerable advantages related to reader use, they have garnered much attention and are being employed in next-generation LFA assays, as introduced in many research papers and reviews. As an example, a multiplexed LFA was established by Cao et al. based on a fluorescence optical reader for simultaneous detection of procalcitonin (PCT) and C-reactive protein in serum with a minimal sample quantity of 27.5 μL that can be achieved using a finger prick. The produced low-cost portable system obtained a linear regression correlation of 0.97 (*p* < 0.01) and 0.95 (*p* < 0.01) for PCT and C-reactive protein, respectively. This system was capable of differentiating between bacterial and viral infections at POC settings within 20 min [71].

**Figure 4:**
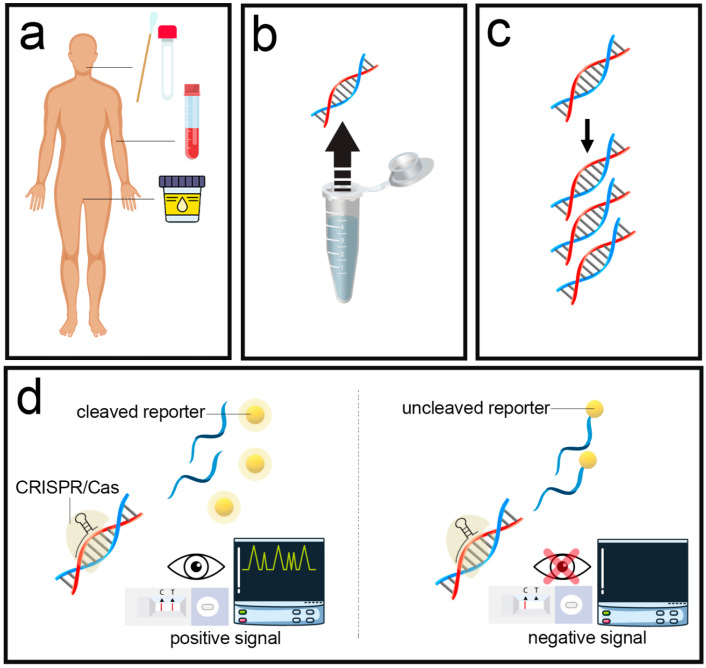
CRISPR/Cas assays for nucleic acid detection of SARS-CoV-2. (**a**) Various types of clinical samples collected from patients. (**b**) RNA is isolated from the sample. (**c**) DNA amplification by isothermal methods. (**d**) SARS-CoV-2 nucleic acid detection: for example, in the positive sample, the CRISPR/Cas complexes attach to the target sequence of the nucleic acid, and the collateral cleavage process starts, followed by cleaving the fluorescence reporter nucleic acids. LFA can be analyzed based on nanolabels using both fluorescence and naked-eye detection. In the negative sample example, the CRISPR/Cas complexes will not attach to the target sequence of the nucleic acid and the collateral cleavage process will not be started.

A clustered, regularly-interspaced short-palindromic repeat (CRISPR)-associated nuclease (Cas) system has recently emerged as a promising diagnostic tool owing to its capability to target specific genes. This diagnostic system employs Cas effector proteins (Cas9, Cas12, and Cas13) as specialized recognition elements that may be applied in combination with a variety of readouts for on-site analysis (Figure 4) [72]. CRISPR-based DNA or RNA diagnostic systems, including the specific high-sensitivity enzymatic reporter-unlocking (SHERLOCK) (using Cas13a) [73] and DNA endonuclease-targeted CRISPR Trans reporter (DETECTR) (using Cas12a, Cas14) [74], are appropriate for LFA-based detection and have been utilized for the diagnosis of viruses in general and SARS-CoV-2 in particular [4,75]. They are compatible with isothermal nucleic acid amplification and simple LF dipstick readout that enables sensitive/specific on-site testing [76]. Isothermal molecular amplification, such as reverse-transcription (RT)-loop-mediated isothermal amplification (LAMP) or RT-recombinase polymerase amplification (RPA), is employed as a first step to boost the assay performance, followed by a CRISPR-based recognition step triggered through sequence-specific detection of target nucleic acids using a guiding RNA (gRNA) or CRISPR RNA (crRNA)-Cas compound [77,78]. Cas12 or Cas13 enzymes are frequently utilized, which collaterally cleave a reporter molecule once activated by attaching to the complementary target sequence. After Cas enzymes activation and cleavage, the oligonucleotide reporter can attach to the test and control regions of nitrocellulose film. Dead Cas9 (dCas9) also attaches target sequences without cleavage activity, leading to colocalization of the dCas9, target oligonucleotide sequence, and nanolabels at the test region [4,75]. Liu et al. presented a CRISPR-based LFA for SARS-CoV-2 using multienzyme isothermal rapid amplification and CRISPR-Cas13a nuclease. The presented LFA achieved a limit of detection of 0.25 copy/μL. They analyzed 52 COVID-19-positive and 101 COVID-19-negative clinical specimens by the CRISPR-based LFA, of which the results showed 100% consistency with real-time polymerase chain reaction (RT-PCR) [38]. There have been many efforts to establish POC SERS devices. For instance, Joung et al. developed a portable SERS LFA based on localized surface plasmon resonance. They examined 54 clinical samples, which obtained 49 positive and 5 negative results. Two false negative results were observed by the SERS LFA device, while with the commercial LFA, 21 false negative results were distinguished. The obtained results with clinical samples show that the SERS LFA device significantly reduced the false-negative rate compared to that of commercial LFA strips [39]. A thermal contrast magnification (TCA) reader was proposed by Wang et al. The reader consists of an emitter for multiple wavelength surface-emitting lasers with an infrared camera for the produced heat reading and software for result detection and processing. This system has eight-fold improved sensitivity compared to naked eyes or colorimetric readers [40]. Sena-Torralba et al. discussed how nanomaterial could address the inherent weaknesses of traditional LFAs and summarized the obstacles toward commercialization and the creation of new hardware as well as a portable reader for LFA quantification and analysis [79]. Deng et al. reviewed the evolving methods to boost the sensitivity of LFAs, and the future viewpoints and limitations in this research area were also discussed [80]. Liu et al. exhaustively discussed approaches for augmenting the sensitivity and specificity of LFAs [69]. In addition to standard reader devices, smartphone readers are also emerging as favorable alternative systems for improving the utility of *POC* tests in which detailed information or details are provided in other studies [11,81,82,83,84]. Using a smartphone, it is possible to quantify the test line color intensity via a camera or a more sophisticated photodetector to estimate the number of target molecules in the specimen. Smartphones have recently played an important role in portable LFA devices to obtain, evaluate, and exhibit measuring signals with high sensitivity [83,85,86,87]. For example, an automated computational imaging system relying on the continuous monitoring of test region intensity, background, control region attendance, and sample flow rates for real-time and, in parallel, evaluating multiple LFAs was introduced by Colombo et al. [88]. The system ran on a smartphone by integrating simple 3D-printed housing and the custom code. By comparing the results from the proposed method with the conventional naked-eye readout, they displayed a shorter time-to-result across various amounts of target analyte and fewer false negatives in a sample with low analyte concentrations. Furthermore, capturing and analyzing images via modern communication technology approaches such as the Internet of Things (IoT), machine learning (ML), deep learning (DL), big data analytics (BDA), artificial neural networks (ANN), artificial intelligence (AI), and smartphones can provide consistent and repeatable color intensity estimations of the test regions, especially for samples with low analyte concentrations [83,89,90,91,92,93].

## 5. Extraction and Enrichment of the Target Molecule in the Sample

The sensitivity and LOD of LFAs can be increased by the target analyte extraction/preconcentration in a specimen, which is commonly present in complex matrices at very low concentrations. The target analyte can be extracted/concentrated before the specimen is injected into the LFA assay or throughout the flow period of LFA. The former was employed by Sharma et al., who utilized antibody-functionalized magnetic beads to concentrate and isolate a target analyte in a specimen prior to LFA and obtained a 10-fold enhancement in sensitivity [41]. This strategy revealed great applicability in the lab; however, the requirement of several washing processes makes it less user-friendly and more prone to error. In the case of concentrating during the flow period of LFA, Kim et al. described a nanoelectrokinetic (NEK)-based method for SARS-CoV-2 immunoglobulin G enrichment from serum samples at specific positions on paper with a balanced electrokinetic force [42]. Experimental results demonstrated that this method enhances the LoD up to 32-fold with an increase in analytical sensitivity (16.4%).

## 6. Conclusions and Future Outlook

The traditional LFA is by far one of the most commercially successful bio-analytical POC devices to achieve on-site analysis of biomarkers. Even though great progress has been achieved in LFA development, there are still a few challenges, such as low clinical sensitivity and specificity, especially for low concentrations of analytes, lack of quantitation, difficulties in multi-analyte detection or multiplexing, and an inability to conduct multi-step analysis, that need to be solved. We reviewed recent efforts on assay improvement and target enrichment of limited samples by preamplification to address the remaining challenges of existing LFAs and make them reliable, accurate, and cost-effective for diagnostics. For instance, assay optimization (by adjusting the capillary flow rate and increasing biorecognition element immobilization on the paper surface using physical and chemical pretreatment) and signal amplification techniques (by new chemical/physical modifications, new label design, and reader use) can improve detection sensitivities and enable one-step multi-analyte detection in LFAs. The analytical specificity of the assay can also be improved through the usage of capture and detection molecules with high affinity and/or avidity. Notably, the preparation of new antibodies with reduced target binding motifs is vital for accomplishing the desired analytical sensitivity and specificity. Moreover, by employing a new nanostructure as the tracing tag for labeling detector molecules or as the immobilization coverage of capture molecules and the Internet of Medical Things as well as artificial intelligence, not only can the signal amplification be improved, but these approaches can also offer promising candidates for the development of reliable and accurate multi-array or multi-step-based LFAs. We expect that, in the future, more development of these and the utilization of ML, DL, BDAs, ANNs, AI approaches, and other signal amplification techniques will be eminent in the development of high-performance, affordable, and digitally-connected LFAs.

## Figures and Tables

**Table 1:** Summary of the different sensitivity and specificity improvement approaches reported for LFA.

Approach	Method/Material	Detection of	Recognition Element	Improvement	Comp. to	Ref.
Assay Improvement						
*Assay optimization*: flow rate decrease	ball pen writing-without-ink	HIV	DNA probe	2-fold	conv. LFA	[24]
	laser-patterned geometric control barriers	PCT	antibody	LOD of 1 ng/mL	conv. LFA	[25]
	apply pressure on the top of the membrane	CRP	antibody	2-fold	conv. LFA	[26]
	trimethylsilyl cellulose barrier	SARS-CoV-2 spike antigen	antibody	9.1- fold	conv. LFA	[27]
	imprinting barricades on the path of flow using water-insoluble ink	human chorionic gonadotropin	antibody	8-fold	conv. LFA	[28]
*Assay optimization*: increasing immobilization efficiency	nitrocellulose nanofibers	human chorionic gonadotropin	antibody	50-fold	conv. LFA	[29]
	graphene oxide	UCH-L1	antibody	LOD of 11 pg/mL, 2–3-fold	no comparison	[30]
	CBP31-BC fusion	SARS-CoV-2	antibody	100% accuracy	RT-PCR	[22]
	CBP31-BC fusion	PSA	antibody	10-fold	conv. LFA	[31]
*Signal amplification*: chemical/physical modifications	Au-Ag alloy nanoparticles on silica surfaces	PSA	antibody	LOD of 0.30 ng/mL	no comparison	[32]
	nanozyme (PtNPs)	TAC	no bioreceptor	no report	no comparison	[33]
*Signal amplification*: new label design	SERS-LFA/GNPs	cardiac troponin I	antibody	78-fold	gold nanoparticle-LFA	[34]
	SERS-LFA/palladium—gold core–shell nanorods/catalytic hairpin assembly (CHA)	Squamous cell carcinoma/miRNA	streptavidin	LOD of fM	RT-PCR	[35]
	plasmonic construct	SARS-CoV-2 S1 antibody	antibody	5675-fold	gold nanoparticle-LFA	[36]
	Silica nanosphere/GNPs/rQDs	H-FABP	gQDs/antibody	LOD of 0.21 ng/mL	Conv. fluorescent LFA	[37]
*Signal amplification: reader use*	CRISPR-Cas13a-LFA	SARS-CoV-2	fluorescein isothiocyanate-secondary antibody	LOD of 0.25 copy/μL	RT-PCR	[38]
	SERS-LFA/GNPs	SARS-CoV-2	antibody	no enhancement	conv. LFA	[39]
	thermal contrast magnification	malaria	antibody	8-fold	colorimetric reader	[40]
Extraction and Enrichment of the Target Molecule in the Sample	magnetic field-assisted preconcentration	cardiac troponin	antibody	10-fold	conv. LFA	[41]
	nanoelectrokinetic (NEK)/preconcentration	SARS-CoV-2-IgG	antibody	32-fold	conv. LFA	[42]
